# Assessment of Maternal Health Literacy and Its Association With Pregnancy Outcomes Among Women

**DOI:** 10.7759/cureus.111405

**Published:** 2026-06-24

**Authors:** Parsa Ahsan, Mahnoor Fatima, Beenish Malik, Asghar Awan

**Affiliations:** 1 Epidemiology and Public Health, University of Lahore, Lahore, PAK; 2 Family Medicine, Bacha Khan Medical College, Mardan, PAK

**Keywords:** antenatal care, maternal health, maternal health literacy, neonatal outcomes, pregnancy outcomes

## Abstract

Background

Maternal health literacy is a key factor in helping women acquire, understand, and utilize health information during pregnancy. Poor health literacy may impede effective decision-making and impact maternal and newborn health. The objective of this study was to assess maternal health literacy and its association with pregnancy outcomes among postnatal women in Lahore, Pakistan.

Methodology

This analytical cross-sectional study was conducted among 374 postnatal women at the University of Lahore, Pakistan, between January 1, 2025, and June 30, 2025. The Maternal Health Literacy and Pregnancy Outcome Questionnaire recorded data, socio-demographic data, and obstetric data. IBM SPSS Statistics software, version 26.0 (IBM Corp., Armonk, NY), was used for statistical analyses. Data did not satisfy the assumption of normality, so nonparametric tests were used to explore associations among the study variables.

Results

Women with live births demonstrated significantly higher maternal health literacy scores than those reporting neonatal deaths (Mann-Whitney U = 214.5, p < 0.001). Pregnancy outcomes were significantly associated with educational status, household income, parity, neonatal birth weight, neonatal intensive care unit (NICU) admission, APGAR score, and maternal comorbidities (all p < 0.05).

Conclusions

Maternal health literacy was positively associated with favorable pregnancy outcomes. Further prospective studies are required to determine whether interventions aimed at improving maternal health literacy can improve maternal and neonatal outcomes.

## Introduction

Maternal health remains a main public health issue, particularly in the middle- and low-income countries where pregnancy and childbirth complications are still a significant contributor to maternal and neonatal mortality and illness. A variable that could affect these results is health literacy, which refers to a person's ability to access, interpret, and understand health information to make health decisions [1]. Women with good health literacy can generally better interact with health services, comprehend health guidance, and practice behaviors that promote healthy pregnancies and safe childbirth experiences [2]. Maternal health literacy (MHL) is defined as the ability of an individual woman to seek out and act on information about pregnancy, birth, and postpartum care [3].

Increased maternal health literacy has been linked with better knowledge of maternal nutrition, improved attendance at antenatal care visits, adherence to prescribed medications, higher immunization uptake, successful breastfeeding practices, and early recognition of obstetric warning signs. However, low maternal health literacy can also negatively impact decision-making, healthcare-seeking, adherence to medical advice and recommendations, and maternal and/or neonatal outcomes [4,5]. Perinatal mortality, neonatal intensive care unit (NICU) admissions, low birth weight, and preterm delivery remain important global health issues [6]. Limited education, socioeconomic disadvantage, limited access to healthcare services, and poor health awareness have been suggested as key factors that influence poor pregnancy outcomes in past studies [6,7]. Difficulty accessing health information and effective health services tends to be a problem for women with lower levels of education [7].

Multiple studies suggest that MHL is associated with safer maternal health behaviors and better pregnancy outcomes [8,9]. These women are likely to have regular attendance at antenatal visits, follow health advice, identify complications early, and access care in a timely manner as needed [9]. Furthermore, growing evidence indicates that MHL is associated with better newborn care practices, such as successful breastfeeding, on-schedule childhood immunizations, and proper utilization of health services after birth [10]. The utilization of maternal health services in Pakistan is influenced by multiple factors, including educational attainment, socioeconomic status, healthcare accessibility, and cultural practices [11]. Despite recent improvements in maternal healthcare services, substantial barriers remain that limit women's access to quality healthcare and reliable health information [11,12]. These challenges may adversely affect maternal health literacy and, consequently, maternal and neonatal outcomes.

Although numerous international studies have reported significant associations between maternal health literacy and maternal or neonatal health outcomes, the available evidence remains heterogeneous. Variations in study populations, healthcare settings, assessment tools, and analytical methods have led to inconsistent findings across different regions [8-10]. Furthermore, many previous studies have focused primarily on maternal health behaviors, antenatal care utilization, or neonatal care practices rather than comprehensive pregnancy outcomes. Evidence from low- and middle-income countries, particularly Pakistan, remains limited, despite the country's unique socioeconomic and healthcare challenges. Therefore, further research is needed to better understand the relationship between maternal health literacy and pregnancy outcomes within the Pakistani context. Such evidence may assist clinicians, healthcare providers, and policymakers in developing targeted interventions aimed at improving maternal and neonatal health outcomes.

Research objectives

To assess maternal health literacy and its association with pregnancy outcomes among women attending a tertiary care hospital in Lahore.

## Materials and methods

Study design and setting

This analytical cross-sectional study was conducted at the University of Lahore, Pakistan, from January 1, 2025, to June 30, 2025.

Sample technique and size

The sample size was estimated using the single-population proportion formula for cross-sectional studies, assuming a 95% confidence level (Z = 1.96), a 5% margin of error, and an anticipated prevalence of poor maternal health literacy of 41.6%. The calculated minimum sample size was 374 participants. Participants who met the eligibility criteria were enrolled using a nonprobability convenience sampling technique.

Participant selection

Women aged 18-45 years who were admitted to the postnatal ward within 24-48 hours after delivery and had delivered at term were eligible for inclusion in the study. Women with known physical or psychological disorders affecting communication or response accuracy, those requiring intensive care management, women with diagnosed mental health disorders impairing comprehension, and participants who were unable to complete the questionnaire due to language barriers, exhaustion, or refusal to participate were excluded from the study.

Data collection procedure

Data were collected through face-to-face interviews using a structured questionnaire that included socio-demographic characteristics, maternal health literacy, obstetric history, and pregnancy outcomes. Maternal health literacy and pregnancy outcomes were assessed using the validated Maternal Health Literacy and Pregnancy Outcome Questionnaire (MHLAPQ), originally developed and validated by Mojoyinola JK [13].

Validity and reliability

The reliability of the questionnaire was evaluated using Cronbach’s alpha coefficient. The maternal health literacy section demonstrated acceptable internal consistency (α = 0.791), while the pregnancy outcome section also showed acceptable reliability (α = 0.822).

Questionnaire scoring and data management

The Maternal Health Literacy and Pregnancy Outcome Questionnaire (MHLAPQ), developed and validated by Mojoyinola JK, was used to assess maternal health literacy and pregnancy outcomes [13]. Responses to the maternal health literacy items were scored according to the questionnaire guidelines, and individual item scores were summed to obtain a total maternal health literacy score. Higher scores indicated greater maternal health literacy. Pregnancy outcome scores were calculated similarly by summing responses to outcome-related items, with higher scores reflecting more favorable maternal and neonatal outcomes.

Prior to statistical analysis, all completed questionnaires were reviewed for completeness and accuracy. Questionnaires containing substantial missing information were excluded from the study. No variable contained sufficient missing data to require statistical imputation; therefore, complete-case analysis was performed. Because maternal health literacy and pregnancy outcome scores were not normally distributed, these variables were summarized as medians and interquartile range (IQR), and non-parametric statistical tests were applied for inferential analyses.

Ethical considerations

Ethical approval was obtained from the Ethical Review Committee of the University of Lahore (UIPH/ERC/2025/041) before the commencement of the study. Written informed consent was obtained from all participants prior to enrollment. Confidentiality and anonymity were maintained throughout the study, and participation was entirely voluntary. Participants were informed of their right to refuse to participate or withdraw from the study at any stage without any consequences.

Data analysis

Data were analyzed using IBM SPSS Statistics software, version 26.0 (IBM Corp., Armonk, NY, USA). Descriptive statistics were used to summarize participant characteristics. Categorical variables were presented as frequencies and percentages, while continuous variables were summarized as medians and interquartile ranges (IQRs) because normality assumptions were not satisfied. Normality of maternal health literacy and pregnancy outcome scores was assessed using the Shapiro-Wilk test. Since both variables demonstrated non-normal distributions, non-parametric statistical methods were employed. Comparisons between two independent groups were performed using the Mann-Whitney U test, while associations between categorical variables were assessed using the Chi-square test. Potential confounding factors, including maternal age, educational status, household income, residence, parity, and maternal comorbidities, were considered when interpreting the findings because these variables may influence both maternal health literacy and pregnancy outcomes. A p-value of ≤0.05 was considered statistically significant.

Study variables

The primary exposure variable was maternal health literacy, measured using the Maternal Health Literacy and Pregnancy Outcome Questionnaire (MHLAPQ). The primary outcome variable was the pregnancy outcome score. Additional independent variables included maternal age, educational status, employment status, husband’s education, household income, residence, family system, parity, mode of delivery, neonatal birth weight, NICU admission, APGAR score, and maternal comorbidities. Several of these variables were considered potential confounders due to their possible influence on both maternal health literacy and pregnancy outcomes.

## Results

**Figure 1:**
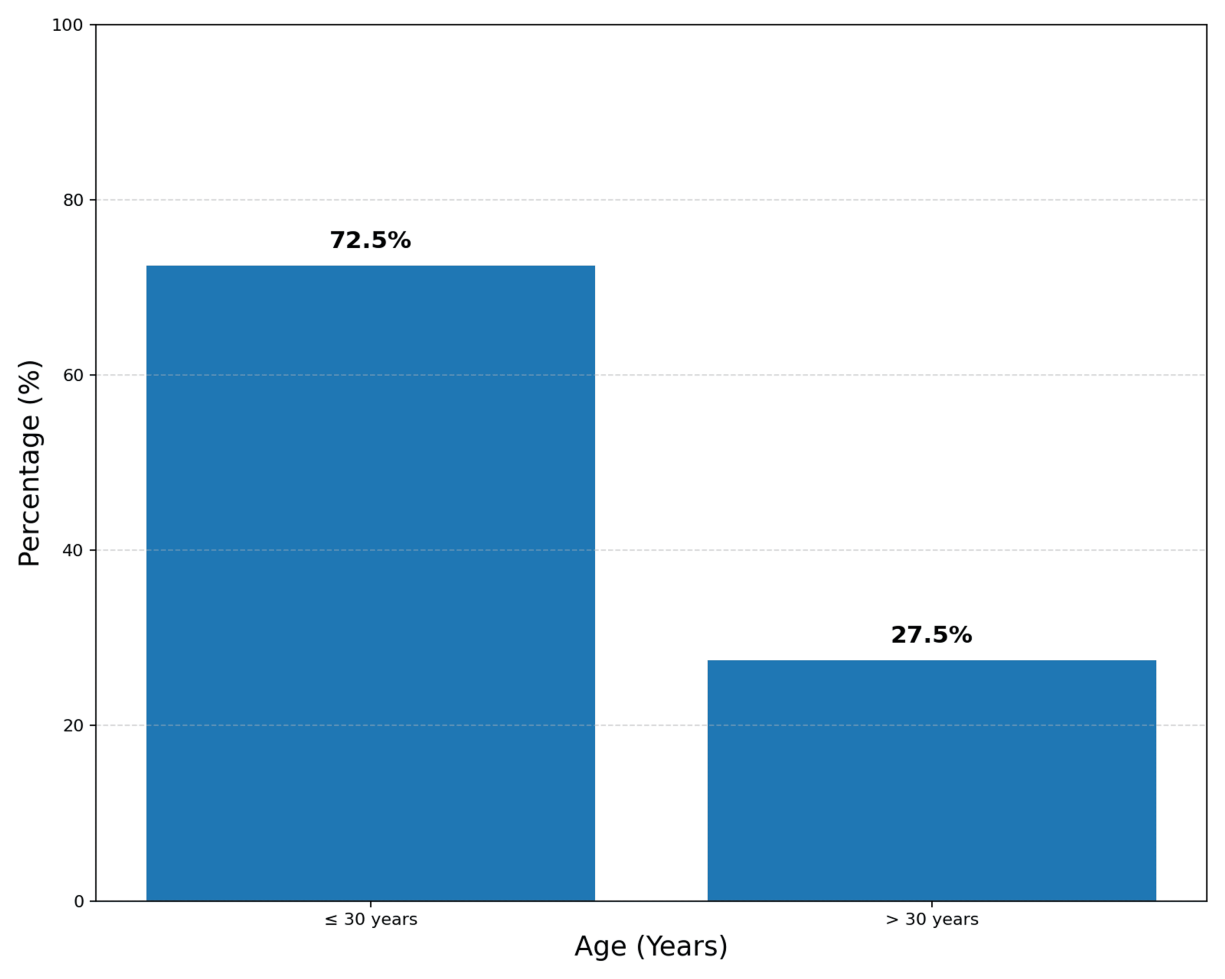
Age distribution of participants (n=374) Distribution of study participants according to age group.

**Figure 2:**
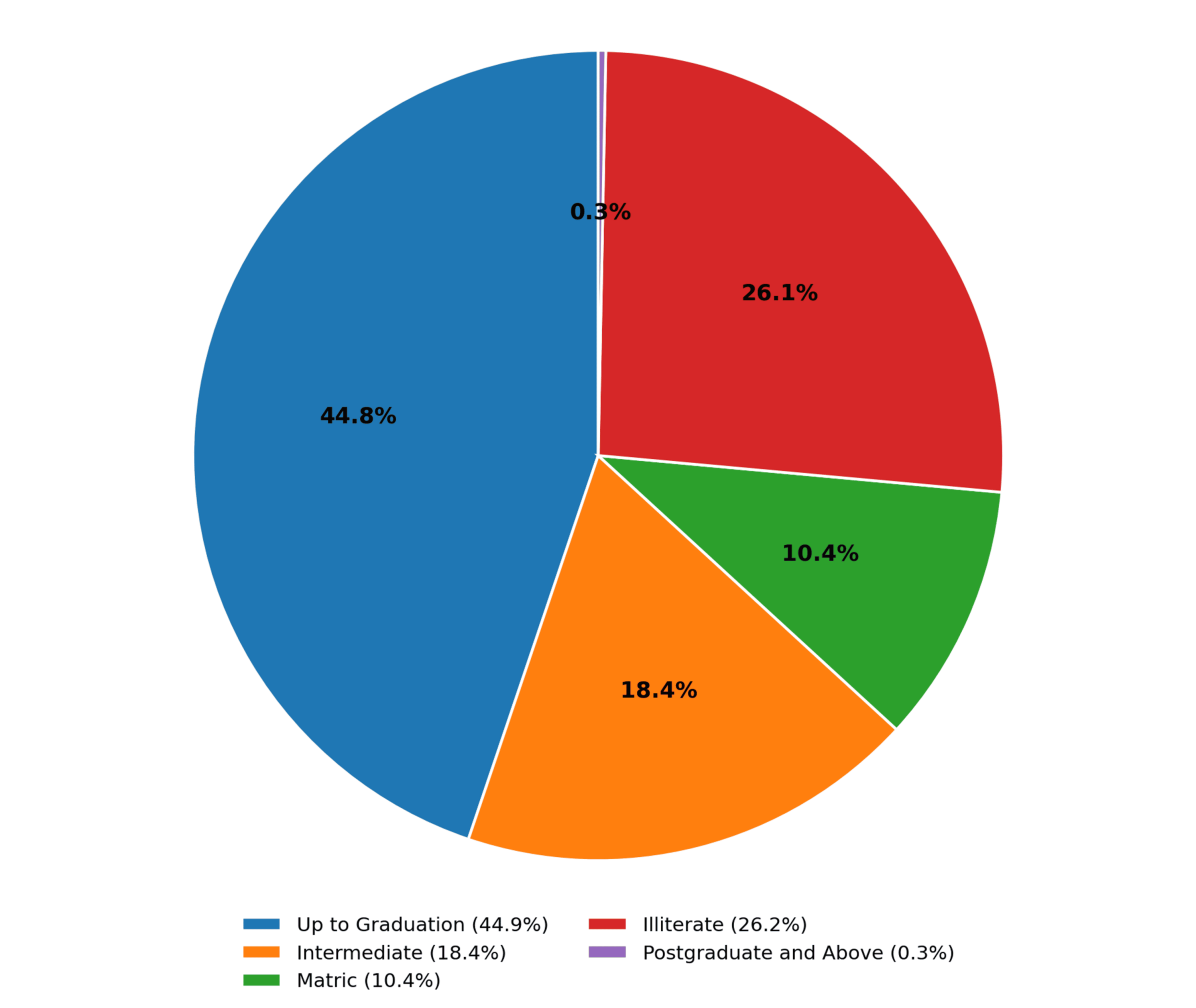
Educational status of participants (n=374) Distribution of study participants according to educational status.

A total of 374 postnatal women were included in the study. The participants represented diverse socio-demographic backgrounds. Detailed socio-demographic characteristics are presented in Table 1. Figure 1 presents the age distribution of study participants, while Figure 2 illustrates their educational status.

**Table 1:** Socio-demographic characteristics of study participants (n=374) Data are presented as n (%).

Variable	Category	n (%)
Age	≤30 years	271 (72.5)
	>30 years	103 (27.5)
Employment Status	Housewife	303 (81.0)
	Working	71 (19.0)
Husband’s Education	Illiterate	59 (15.8)
	Literate	315 (84.2)
Monthly Household Income	≤50,000 PKR	102 (27.3)
	>50,000 PKR	272 (72.7)
Residence	Urban	200 (53.5)
	Rural	174 (46.5)
Family System	Nuclear	144 (38.5)
	Extended	230 (61.5)

Normality assessment demonstrated that maternal health literacy and pregnancy outcome scores were not normally distributed; therefore, non-parametric statistical tests were used for inferential analyses (Table 2).

**Table 2:** Normality assessment of maternal health literacy and pregnancy outcome scores (n=374) Data are presented as Shapiro-Wilk statistics and p-values. Statistical significance was considered at p<0.05.

Variable	Shapiro–Wilk Test Statistic	p-Value
Maternal Health Literacy (MHL) Score	0.857	<0.001
Pregnancy Outcome Score	0.222	<0.001

The obstetric and clinical characteristics of the participants are summarized in Table 3, while maternal health literacy and pregnancy outcome scores are presented in Table 4.

**Table 3:** Obstetric and clinical characteristics (n=374) Obstetric and clinical characteristics of study participants (n=374). Data are presented as n (%).

Variable	Category	n (%)
Gravidity	Primigravida	72 (19.3)
	Multigravida	302 (80.7)
Mode of Delivery	Vaginal Delivery	234 (62.6)
	Cesarean Section	140 (37.4)
NICU Admission	Yes	89 (23.8)
	No	285 (76.2)
Birth Weight	<2.5 kg	110 (29.4)
	≥2.5 kg	264 (70.6)
APGAR Score	>7	299 (79.9)
	≤7	75 (20.1)

**Table 4:** Maternal health literacy and pregnancy outcomes Data are presented as median (IQR).

Variable	Median (IQR)
Maternal Health Literacy (MHL) Score	40 (22.5–56.0)
Pregnancy Outcome Score	32 (29.0–33.0)

Women with live births had significantly higher maternal health literacy scores than those who experienced neonatal deaths (Mann-Whitney U = 214.5, Z = -4.82, p < 0.001). Maternal education was significantly associated with pregnancy outcomes (χ² = 12.7, df = 2, p = 0.003). Significant associations were also observed between pregnancy outcomes and husband’s education, residence, household income, parity, neonatal birth weight, NICU admission, APGAR score, and maternal comorbidities (p < 0.05). Lower educational attainment, higher parity, adverse neonatal indicators, and maternal comorbidities were associated with less favorable pregnancy outcomes. These findings suggest that maternal health literacy and socio-demographic characteristics play important roles in influencing maternal and neonatal health outcomes.

## Discussion

The present study examined the correlation between MHL and pregnancy outcomes in postpartum women who visited a tertiary health care center in Lahore, Pakistan. The results showed that maternal health literacy is associated with better maternal and neonatal outcomes among women with high literacy levels [13]. These observations suggest that social and educational advantages may facilitate better access to healthcare information and improve the utilization of maternal health services [14,15]. The study further demonstrated that obstetric and neonatal characteristics such as parity, neonatal birth weight, NICU admission, APGAR score, and maternal comorbidities were significantly associated with pregnancy outcomes. Mothers with lower levels of maternal health literacy were also more likely to experience adverse neonatal outcomes, including low birth weight and poorer APGAR scores, findings that are consistent with previous literature [16,17].

Women with higher maternal health literacy may be more likely to recognize pregnancy-related warning signs, seek appropriate healthcare services, and engage in recommended maternal health practices. However, because of the cross-sectional nature of this study, it was not possible to determine whether higher maternal health literacy directly leads to improved pregnancy outcomes. The observed relationships should therefore be interpreted as associations rather than causal effects [18,19]. The current results corroborate previous international studies that found a correlation between the level of maternal health literacy and the utilization of preventive health care services and healthy maternal behaviors during pregnancy [20].

Well-informed women are more likely to have confidence in making decisions about their maternal health, in following recommended clinical advice, and in communicating with health care providers. These factors may contribute to favorable pregnancy experiences and neonatal outcomes; however, causal relationships cannot be established from the present cross-sectional study [20,21]. Maternal health literacy is influenced by several social and environmental factors. Having a good education, cultural norms, economic resources, and healthcare accessibility all impact a woman's ability to effectively use and gain health information [22]. These women in underserved or resource-poor communities might face barriers to accessing quality maternal health education and healthcare services. Thus, efforts to enhance maternal health outcomes must focus on both personal knowledge and social determinants of health [22,23].

The findings of this study suggest that integration of structured maternal health education into routine antenatal care services may be beneficial; however, prospective studies are needed to determine its effectiveness. Healthcare providers should educate women, in a culturally appropriate and understandable manner, Healthcare providers should educate women, in a culturally appropriate and understandable manner, about nutrition, medication use, danger signs in pregnancy, breastfeeding, and infant care., medication use, danger signs in pregnancy, breastfeeding, and infant care [2]. Additionally, community outreach and public health approaches specifically targeting women from socioeconomically disadvantaged populations can further enhance maternal health literacy and help mitigate preventable maternal and neonatal complications [2,24]. Although significant associations were identified between maternal health literacy and pregnancy outcomes, residual confounding cannot be excluded. Factors such as family support, healthcare accessibility, cultural beliefs, health-seeking behavior, and other unmeasured socioeconomic influences may affect both maternal health literacy and pregnancy outcomes. Furthermore, reverse causation is possible, whereby women who experience more favorable pregnancy outcomes may subsequently demonstrate greater health literacy and engagement with healthcare services. Prospective longitudinal studies are required to better establish temporal relationships and clarify potential causal pathways.

Limitations

Several limitations should be acknowledged. First, the cross-sectional design prevents determination of temporal or causal relationships between maternal health literacy and pregnancy outcomes. Second, the use of convenience sampling and recruitment from a single tertiary care institution may limit the generalizability of the findings to other populations within Pakistan. Third, self-reported questionnaire responses may be subject to recall and reporting bias. Fourth, multivariable regression analysis was not performed; therefore, the independent association between maternal health literacy and pregnancy outcomes, after adjustment for potential confounding factors such as educational status, household income, parity, and maternal comorbidities, could not be determined. Finally, residual confounding from unmeasured variables cannot be excluded.

## Conclusions

Maternal health literacy was positively associated with favorable pregnancy outcomes among postnatal women in Lahore, Pakistan. Educational status, household income, parity, maternal comorbidities, and neonatal clinical indicators were also significantly associated with pregnancy outcomes. These findings highlight the potential importance of maternal health literacy as a factor related to maternal and neonatal health. However, due to the cross-sectional nature of the study, causal relationships cannot be established. Further prospective, multicenter studies are required to determine whether interventions aimed at improving maternal health literacy can contribute to improved maternal and neonatal outcomes.
